# Using design-based research to develop a Mobile Learning Framework for Assessment Feedback

**DOI:** 10.1186/s41039-018-0070-3

**Published:** 2018-04-20

**Authors:** Mireilla Bikanga Ada

**Affiliations:** School of Engineering and Computing, University of the West of Scotland, High St, Paisley, UK

**Keywords:** Design-based research, Mobile learning, Assessment feedback, Framework

## Abstract

Students’ lack of engagement with their assessment feedback and the lack of dialogue and communication for feedback are some of the issues that affect educational institutions. Despite the affordance that mobile technologies could bring in terms of assessment feedback, research in this area is scarce. The main obstacle for research on mobile learning assessment feedback is the lack of a cohesive and unified mobile learning framework. This paper thus presents a Mobile Learning Framework for Assessment Feedback (MLFAF), developed using a design-based research approach. The framework emerged from the observation of, and reflection upon, the different stages of a research project that investigated the use of a mobile web application for summative and formative assessment feedback. MLFAF can be used as a foundation to study the requirements when developing and implementing wide-scale mobile learning initiatives that underpin longitudinal practices, as opposed to short-term practices. The paper also provides design considerations and implementation guidelines for the use of mobile technology in assessment feedback to increase student engagement and foster dialogic feedback communication channels.

## Introduction

Both student learning and student satisfaction are affected by assessment and feedback—two crucial elements of student experience. In fact, feedback has been recognised as one of the most crucial aspects which helps develop student learning (Black and Wiliam 1998). However, even after years of research and the increased adoption of technology to provide feedback, the assessment and feedback area is still a source of concern and continues to have a lower satisfaction rating than other areas in the National Student Surveys (NSS) (Boud and Molloy 2013). Meanwhile, the ubiquitous nature of mobile handheld devices is making mobile learning an attractive option in education. Mobile learning research has provided evidence that it can enhance, extend and enrich the concept and activity of learning itself (Traxler 2011), as mobile devices can ‘support every pedagogic option, including the didactic and the discursive, the individual and the social’ (Traxler 2010). However, the use of mobile learning in the educational sphere is still quite low as compared to other areas of a mobile user’s life. Mobile learning initiatives have focused in many areas of education, and different categories of mobile learning pedagogy are at different stages of development and research. A literature review by Bikanga Ada (Interrelationship between pedagogy, theories, objectives, and features: mobile learning design, n.d.) highlights the fact that many studies are still investigating attitudes towards mobile learning, indicating its level of infancy. One of the areas that is still in its infancy is assessment feedback in relation to mobile learning.

### Background and literature review

#### Assessment feedback

The importance of assessment and feedback in student learning has been explored in detail in previous literature. Reports and surveys (Ferrell 2012; NSS 2012) highlight student dissatisfaction with the assessment and feedback system. This ‘troublesome issue’ (Nicol et al. 2014, p. 102) has long been a challenge for both staff and students even before the introduction of the National Student Survey (NSS) and remains the weakest factor in NSS (Bell and Brooks 2017). Evans (2013) reviewed 460 articles on assessment feedback from 2000 to 2012 and found that most of the problems arise due to the fact that the student population has increased in higher education, due to which the unit of resource is being stretched and there is growing pressure on academic staff regarding traditional assessment. Bikanga Ada et al. (2017) investigated students’ (*n* = 540) and educators’ (*n* = 70) perception of assessment and feedback, and identified several issues that have also been highlighted in the literature. These issues include lack of student engagement and motivation with assessment feedback; educators being unhappy because, despite all their effort, students do not collect their assessment feedback; and high student numbers affecting the capacity to provide timely and personalised feedback (Bikanga Ada et al. 2017). Generally, formative and summative assessments are used to evaluate students. Formative assessments are tasks or activities that provide feedback to students on their learning, while summative assessments are used to evaluate students at the end of a course or a module. Assessment feedback is feedback provided to students upon completion of either a formative or a summative assessment. Despite the fact that many educational institutions’ policies require feedback to be ‘an interface between teachers’ pedagogical goals; students’ learning needs; and institutional and governmental education policies, which structure and regulate practices and procedures’ (Bailey and Garner 2010, p.188), there is a decline in the practice of offering feedback to students (Charles et al. 2011). Providing feedback to students plays a vital role in increasing student achievement. Its ease of access has been identified as one of the aspects students value the most (Hepplestone et al. 2016), and one of the ways in which this access is facilitated is when the feedback is online. The potential for technologies to help educators provide feedback that is more personal and rich has been highlighted in various publications (Belshaw 2010). Crook et al. (2012) note that technology ‘provide[s] the innovative edge that can help students engage more effectively with their feedback’ (p. 387). A literature review (Interrelationship between pedagogy, theories, objectives, and features: mobile learning design, n.d.) highlights that despite various technologies, including audio, video, screencast and podcast, being used to alleviate the issues in assessment feedback, a larger class cohort still poses some problems to educators. These include ‘extra workload, the inability to provide personalised and individual feedback, and the lack of synchronous or asynchronous communication or dialogue’ (Interrelationship between pedagogy, theories, objectives, and features: mobile learning design, n.d.).

#### Mobile learning

Mobile devices can be the solution to increase students’ access to their assessment feedback. Indeed, mobile technology fosters mobile learning, which ‘accommodates and supports personal agency of the learner in a way that the learner can decide when, where and how he or she will learn; as such, mobile learning is instrumental in just in time and on-demand learning’ (Khaddage et al. 2016, p. 16). Moreover, the affordances of mobile technology include portability, data gathering, communication, interaction with the interface, contextual and active learning, outdoor environment, multimedia creativity and the control of other devices (Parsons et al. 2016). Furthermore (Sung et al. 2016), mobile devices can also be used as tools for stimulating motivation, strengthening engagements and delivering content.

However, Pimmer et al.’s (2016) systematic review of empirical studies on mobile and ubiquitous learning shows that 20 years of mobile learning research has provided little systematic knowledge on the use of mobile technology in different educational designs and the associated educational effects in higher education settings. Furthermore, in the past decade, many studies using student-owned mobile handheld devices have focused on the concept of text messaging (Gemmell et al. 2010; Harley et al. 2007; Naismith 2007; So 2016; Timmis 2012). Despite the growing research involving mobile devices and the social media applications they encompass (Ahern et al. 2016; Gan and Balakrishnan 2017; Ledford et al. 2015; So 2016), and despite the knowledge that mobile learning brings new opportunities for feedback provision (Gaved et al. 2013), the literature on empirical research about using students’ mobile handheld devices for assessment feedback is relatively small. Dearnley et al. (2008) explored the feasibility and identified the issues related to using mobile technologies in the assessment of health and social care students in practice settings. The impact of the mobile devices on the assessment processes and outcomes was positive in general, despite students resenting the use of these devices as they did not belong to them. Another study in which students did not like the devices provided by the university was conducted by Taylor et al. (2010). They introduced mobile learning into health and social care (H&SC) practice placement learning and assessment. The focus of the study was on mobile assessment and a variety of sources of formative feedback that fostered student reflection and deducing further action to improve performance. Dann and Allen (2013) studied the use of iPhone for providing formative assessment to students and other stakeholders within the educational sector. The study shows that feedback that is available on the web and is accessible using mobile devices can increase the opportunity for reflection at a convenient time. Campbell and Morrison (2007) developed a web-based content management system called *Just in Time Medicine* (JIT), an assessment and feedback tool that captured learner progress related to hundreds of clinical skills. The study involved 367 medical students. This study showed that smartphones and tablets are useful in the process of medical learning and technical assessments. However, the feedback associated with their grades was generated automatically and limited to ‘Well done’, ‘Needs improvement’, or ‘Not done/unsatisfactory’. Soh and Ho (2014) studied the use of mobile Applications (m-Apps) by 80 students in a private university in Malaysia to provide dialogic feedback on students’ writing tasks. m-Apps enabled students’ access to the learning material using their smartphone. The positive outcomes of this study include the flexibility of accessing the learning material anytime and anywhere, besides adding to the usefulness, comprehension and transferability of the learning material (p.46).

The limited use of mobile learning within the area specific to assessment feedback is not surprising. Kukulska-Hulme et al. (2011, p. 19) note that mobile technologies will not necessarily be readily adopted for learning, as there are a variety of barriers to adoption. Furthermore, the popularity of mobile handheld device among students and their familiarity with the device does not necessarily make mobile learning attractive (Merchant 2012). A critical issue is the pedagogical integration of technological tools into the curriculum (Kong 2015). Educators need to incorporate ways of leveraging the flexibility of boundary crossing to enhance learning across a multitude of contexts (Schuck et al. 2017, p. 128) by ensuring that both instructional materials and delivery methods are put into a mobile format and remain flexible in different usage environments and situations.

One of the barriers to wide-scale adoption is the lack of confidence educators have in their abilities to use mobile learning strategies. They need to be explicitly guided and supported to adopt these approaches (Parsons et al. 2016). For example, there is a need to develop methods and strategies to generate examples of how to relate and link learning across contexts (Khaddage et al. 2015, p. 627). Additionally, the diversity of mobile learning initiatives and mobile learning frameworks presents a real challenge in higher education (Wishart and Green 2010), including the lack of ‘transferable design framework’, ‘evaluation of the projects’ and ‘explicit underlying pedagogical theory’ (Cochrane, 2014, p. 67). This has led to many researchers highlighting the need for a unified framework (Sølvberg and Rismark 2012) that supports the ecology of mobile learning (Khaddage et al. 2015) and can guide effective instructional design and evaluate the quality of programmes that rely significantly on mobile technologies (Park 2011).

Many frameworks have been designed to address many aspects of mobile learning in general (Crook et al. 2012; Kearney et al. 2012; Koole 2009; Laurillard 2007; Motiwalla 2007; Ozdamli 2012; Park 2011; Parsons et al. 2007). These frameworks share the same overarching themes of pedagogy, learner, context, content, time, social interactions, usability, device and culture, but none of them has a particular focus on assessment feedback. In their literature review on existing mobile learning models and frameworks, Hsu et al. (2014) observed that most frameworks and models focused on ‘pedagogies and learning environment design’. This category of frameworks highlights practical checklists to guide educators to identify the components that serve as a foundation in their mobile learning initiatives. Although the second most common type of frameworks and models, ‘platform/system design’, also involves pedagogies and learning environment design, the focus is generally on the technical aspects. The authors identified only one framework that focuses on evaluation (Vavoula and Sharples 2009), highlighting the scarcity of such frameworks. A framework for evaluating mobile learning should help evaluate each phase of the mobile learning initiative. The framework presented in this paper encompasses these three categories.

To summarise, despite the affordance that mobile technologies could bring in terms of assessment feedback, research on use of mobile learning for assessment feedback is scarce. The main obstacle in adopting mobile learning is the lack of a cohesive, unified mobile learning framework. Considering all the above limitations, the research reported in this paper proposes to address some of the limitations by developing a Mobile Learning Framework for Assessment Feedback. The framework will serve as a guide through the design, development and implementation of the mobile learning initiatives besides being used by educators to evaluate mobile learning outcomes.

### Purpose of the study

**Fig. 1:**
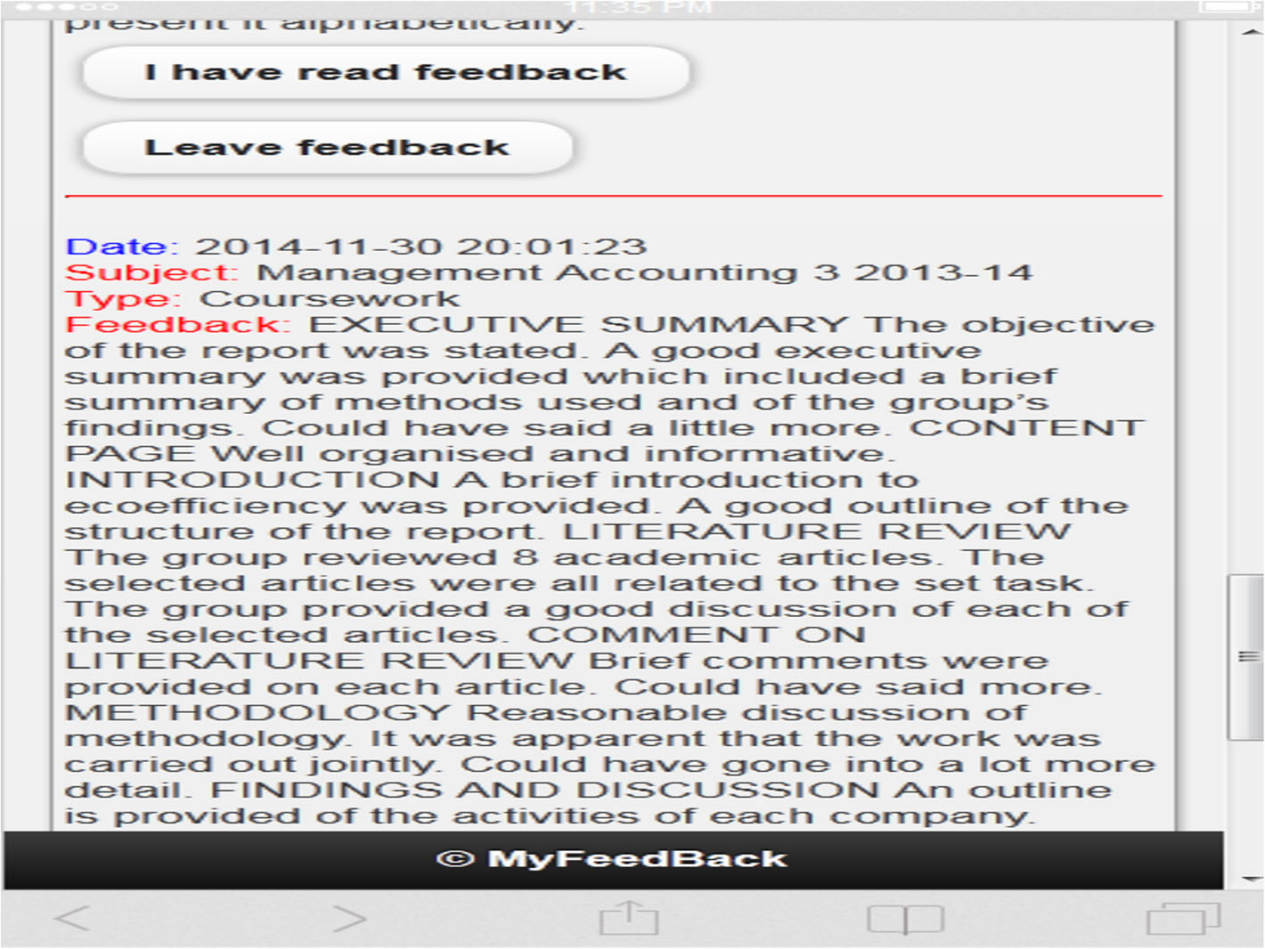
Example of group feedback on a student’s feedback page (Bikanga Ada 2020)

The purpose of the study was to develop a Mobile Learning Framework for Assessment Feedback (MLFAF) through observation of, and reflection upon, the different stages of a research project that uses a mobile web application for assessment feedback. As part of the project, a mobile web application, MyFeedBack, was developed, which enabled the personalisation of group feedback. Group feedback and the associate grades were uploaded once and kept in each student’s account. They were then modified to reflect their individual contributions to the group coursework, where applicable, and then posted to the students’ feedback page. The students would then need their login details to view their own feedback. Uploading just one feedback and making a few changes to the same feedback made the process of feedback provision faster than writing and uploading each feedback individually. Figure 1 (Interrelationship between pedagogy, theories, objectives, and features: mobile learning design, n.d.) shows an example of group feedback as seen on a student’s feedback page. MyFeedBack also enabled monitoring of feedback access and allowed students access to feedback using any device, including mobile handheld devices. A full description of the application is available online (Bikanga Ada 2013a, 2014a). Results reported in this paper focus only on presenting the development phases and evaluation of the framework.

The study explored the following research question:

- ‘What design principles and framework should educators follow in creating mobile learning initiatives, and what pedagogical strategies can best be deployed to enhance student engagement with their assessment feedback and foster dialogic feedback communication channels between educators and students?’

## Method

### Design-based research

The research study is set within the overall framework of design-based research. Wang and Hannafin (2005) defined design-based research as ‘a systematic but flexible methodology aimed to improve educational practices through iterative analysis, design, development, and implementation, based on collaboration among researchers and practitioners in real-world settings, and leading to contextually-sensitive design principles and theories (p. 6)’. The use of design-based research methodology in educational contexts has increased over the past decade and mostly with educational technology innovations and interventions (Anderson and Shattuck 2012, p.25). Its increased use is in response to some traditional research methodologies failing to link theory and practice within the educational practice (Alghamdi and Li 2013, p.3). Design-based research is conducted in real-world contexts (educational settings) due to the complexity of the problems it addresses (Hsu and Ching 2015, p.31). It gives researchers and practitioners the opportunity to produce tools, approaches, theories and products that have been tested in the field and are effective (McKenney and Reeves 2012). The first reason a design-based research approach was adopted in this study is because this research study was situated in real educational context (assessment feedback and mobile learning in higher education). The second reason is the use of new technologies such as student-owned mobile devices and a mobile web application. Recognising the important role of technology in shaping education is fundamental to design-based research. In fact, Amiel and Reeves argue that ‘if we persist in believing in education and technology as value-free, we should not attempt to engage in design-based research and should instead resign ourselves to perpetuating research that effects no systematic change’ (2008, p. 37). With the main focus being on the design and testing of the interventions in order to provide a possible solution to the problem of assessment feedback provision in large classes and lack of student engagement with that feedback, the third reason for adopting a design-based approach was to produce a Mobile Learning Framework for Assessment Feedback (MLFAF) and guidelines.

The study used McKenney and Reeves’ (2012) generic model for design research (GMDR), another name for design-based research, which has three main phases: analysis and exploration, design and construction, and evaluation and reflection. Design-based research follows a cyclic process containing cycles of analysis, design, evaluation and revision that enable the improvement of the interventions over time. Following these iterative cycles, the GMDR phases connect with ‘ongoing practice as the intervention is adopted, enacted, and sustained (implementation) in a particular educational setting while information about the intervention is disseminated and diffused to a wider audience.’(Kukulska-Hulme et al. 2011).

### Participants, settings and design

The research project that enabled the development and evaluation of the MLFAF was conducted in a university in the UK. The focus of the learning activities involved summative and formative assessment feedback. Following the phases of GMDR, the study started with a literature review, followed by initial fact-finding studies involving both lecturers and students. The purpose of the fact-finding studies (survey and follow-up interviews) was to seek participants’ views in relation to assessment feedback and mobile learning (Bikanga Ada et al. 2017). The first draft of the framework emerged from the literature review and was updated after the initial (explorative) fact-finding studies. These corresponded to the analysis/exploration phase of GMDR.

The second phase of the study was concerned with the design and development of a mobile web application, ‘MyFeedBack’. The features of MyFeedBack enabled educators to personalise group feedback and monitor student feedback access, and enabled student access to the feedback using any device, including their mobile handheld devices (Bikanga Ada 2014a). This phase also included early trials of the application, which contributed to the identification of the issues that influenced students’ use of their own devices for mobile learning (Bikanga Ada 2013b), and the investigation of whether students’ grades influenced their decision to access feedback (Bikanga Ada 2014b). This phase, which corresponds to the design/construction phase of the GMDR, triggered further updates of MLFAF.

The last phase was concerned with the evaluation of three studies. In study 1 and 3, students’ summative assessment results (feedback and grades) were uploaded on MyFeedBack, and students in study 2 received only their formative assessment feedback. Participants in study 1 were from the Schools of Health, Nursing and Midwifery (HNM) and Business (*n* = 218). Those in study 2 came from the School of Computing (*n* = 79), and students in study 3 were from the Business School (*n* = 148). While results from observation and digital footprinting from the studies have been disseminated (Bikanga Ada 2014c; Bikanga Ada and Stansfield 2017), the publication reporting on the quasi-experiments’ results, which were positive, is currently under review. The final version of the framework emerged from this phase, which corresponds to the evaluation/reflection phase of the GMDR. Here, the framework went through iterative evaluation, reflection and revision cycles of the individual study, spanning 3 years.

The next section reconstructs the development and evaluation phases of the framework and briefly explains the context surrounding each update of MLFAF.

## Results and Discussion

### Mobile Learning Framework for Assessment Feedback design and evaluation cycles

#### Initial draft based on the literature review

The initial draft of MLFAF had its foundation in previous mobile learning frameworks and the literature review. It brought forward various aspects that have been identified as crucial in mobile learning, including ownership, context, pedagogy, communication and dialogue, and exploration. These aspects of MLFAF have been selected because, altogether, they emphasise the idea of crossing the current boundaries that have existed for so long in educational institutions, thus enabling realms of unprecedented types and levels of engagement and connectivity in a student-centred environment.

##### Ownership

Ownership, which is a key motivational feature (Jones et al. 2006), is about learning in a student-centred environment (Barbara 2010) where students are empowered to make their own decisions facilitated by their own devices. In this learning environment, students can address their own learning interests and needs (Nanney 2004, p. 1). However, the aspect of ownership, which is at the core of this initial draft of the framework, is not only about ownership of the devices, but also encompasses some of the other dimensions that emerged from previous frameworks and literature as contributing factors to mobile leaning (Deng and Tavares 2013; Jones and Issroff 2007; Jones et al. 2006; Kearney et al. 2012; Koole 2009; Laurillard 2007; Park 2011; Parsons et al. 2007). It is about students reclaiming control over the devices that suit their taste or convenience and suit the time they want to access their content, where they want to access that content and the pace at which they interact with it. It is about student motivation and being in control of their choices in a student-centred environment.

##### Pedagogy

Another important aspect, pedagogy, which is defined as ‘any conscious activity by one person to enhance learning in another’(Mortimore 1999, p. 17), has been mentioned in mobile learning frameworks (Ozdamli 2012). One such pedagogical activity is *personalisation*, which is a key aspect identified in many mobile learning frameworks. For example, it enables students to self-control their learning process (Kearney et al. 2012) and to control the content (Motiwalla 2007). Also, personalisation of the content, for instance, requires that educators ensure that the assessment feedback is personalised, reflecting individual student contribution. Furthermore, the benefits of personalised and individual feedback have been highlighted in the literature (Ferguson 2011; Taylor and Burke da Silva 2014). Based on these findings, the pedagogy aspect was added to the framework.

##### Communication and dialogue

Many frameworks (Kearney et al. 2012; Koole 2009; Laurillard 2007, 2009; Motiwalla 2007; Park 2011) have highlighted communication and dialogue as key factors in education. With regard to assessment feedback, the literature emphasises on the importance of dialogue between learners and teachers. For instance, communication issues in feedback have been greatly reported due to the tendency of feedback being a monologue process from teachers to students (Bloxham and Campbell 2010). The conversation theory (Laurillard 2007, 2009) advises that successful learning needs constant two-way conversations and interactions between the educators and learners, and between learners. Communication and dialogue are about building those links between learners and teachers. In light of these findings, *communication and dialogue* were also identified as vital aspects of the Mobile Learning Framework for Assessment Feedback.

##### Context

Another aspect that appears in some frameworks is *context* (Kearney et al. 2012; Koole 2009; Parsons et al. 2007). Despite its importance, many mobile learning frameworks and models have failed to discuss this aspect (Imtinan et al. 2013). Kearney et al. look at context as an expansion of learning in the real world and community. In Koole’s framework, the context of information is what influences mobile learning experiences. In this study, this context of information refers to the context of feedback and access to feedback in authentic contexts. Furthermore, each dialogic communication link between the educator and the student is set within a one-to-one, personalised and individual context, where ideas are negotiated through synchronous or asynchronous exchanges. Context is, therefore, another critical aspect of the framework.

##### Exploration

There is a need for greater understanding of the problems in assessment and feedback practices. Li and De Luca (2014) recommend the starting point be investigating the assumptions and beliefs of stakeholders in assessment practices. It is therefore essential to understand the university’s culture of assessment and feedback and the use of technology-enhanced assessment feedback, investigate whether issues identified in the literature are the same within the institution and investigate the institution’s policies to identify the problems that enhance or hinder the adoption of assessment and feedback policy and its implementation. This dimension is called *assessment and feedback culture and policy*.

On the other hand, literature has identified mobile learning as a key element in the transformation of education. However, literature also indicates that students may be unwilling to use a device that does not belong to them (Dearnley et al. 2008; Taylor et al. 2010) or may not want to use their own device (Franklin 2015). Therefore, students’ own choice to participate in mobile learning using a device of their choice could foster engagement in mobile learning activities (Ferreira et al. 2013). Nonetheless, it is essential to investigate the level of device ownership. This dimension is called *device ownership*.

It is also important to identify whether users would want to use the application (Lazar et al. 2010). Before introducing any form of mobile learning, students’ perceptions of mobile learning need to be investigated or their readiness considered (Cheon et al. 2012; Corbeil and Valdes-Corbeil 2007), as learners may not be willing to accept it (Wang et al. 2009). Also, there is a need to implement ways to find out if and how students make use of the provided feedback (Price et al. 2010) to understand the feedback culture. This dimension is called *willingness and attitude*. It is equally important to investigate whether current technology has the potential to enable the mobile learning activities planned or to help solve the identified issues such as those identified in this research. This dimension is called *IT infrastructure*. All these four dimensions justify the *exploration* aspect in this initial framework.

#### Framework updates based on fact-finding studies

The aspect of *exploration*, which suggested looking into various aspects, including assessment and feedback culture and policy, IT infrastructure, device ownership and attitudes/willingness, was tested using initial fact-finding studies in a university (Bikanga Ada et al. 2017). Findings consolidated the importance of the aspects of the framework in which ownership has a central place as it enables students to use their devices anytime and anywhere for everything, including learning. Thus, technology is seen as a culture, and institutions should tape into these affordances to connect with their students.

The following section describes the changes that were made to the first draft of the framework as a result of the findings from the initial fact-finding studies.

##### Pedagogy (updated)

- Mobile learning boundaries – The results from fact finding revealed that the teaching staff feared being ‘always connected’. It is therefore important to set the boundaries before initiating any mobile learning activities. Students should also be informed when to expect a mobile learning activity and when to contact their lecturer on the mobile learning environment to eliminate the teaching staff’s fear of having to be ‘always connected’.
- Feedback culture change – Results revealed the need to educate both students and tutors on feedback. For example, there were two educators who did not know what feedforward was. On the other side, some students did not recognise feedback. Educators need to reinforce the importance of feedback in student learning enhancement and subsequently for the institution as a whole. Tools such as MyFeedBack or similar that enable the change in students and educators’ feedback culture should be provided.

##### ICT infrastructure

The findings also enabled the enunciation of the pedagogical and technical requirements for a mobile web application (Bikanga Ada 2013a, Bikanga Ada 2014a, 2014b, 2014c). Based on this, another aspect, *ICT infrastructure*, was added to the framework.

#### Framework update based on application development and early trials

Based on the infrastructure requirements identified that led to the addition of the *ICT infrastructure* aspect, a mobile web application, MyFeedBack (Bikanga Ada 2013a, Bikanga Ada 2014a, 2014b, 2014c), was developed. As a result of MyFeedBack development and early trials (Bikanga Ada 2013b, p. 2495), the following dimensions were added to the framework:

##### Context (updated)

- Curriculum – The findings from early trials showed that for a successful integration of mobile learning using student devices, the integration of mobile learning technology into the course design is necessary. The relevance of the mobile learning activities is crucial as the trials highlighted the lack of engagement by students in mobile learning when the topic was not relevant.

##### Pedagogy (updated)

- Participation – The right of educators or students to not participate should be respected. However, to ensure uptake of mobile learning, educators’ participation is crucial and strongly recommended. It was observed that some students did not participate because their lecturer was not using MyFeedBack, hence the importance of interactivity.
- Choice and flexibility – Enable participants to make their own decisions (anytime, anywhere and using any device). Students used different devices during the trials. One crucial point was the shift of paradigm which requires considering the students as the pivotal point of learning. A student-centred learning therefore consists of the need to give students the choice and freedom to use any device to access their learning material and remove the exclusive focus on student-owned mobile handheld devices, which students may not be willing to use (Franklin 2015). Therefore, lecturers need to implement activities that enable flexibility and choice, activities that are ‘device-agnostic’ (Koole et al. 2010).

##### ICT infrastructure (updated)

- Digital education – Both lecturers and students had some difficulties using the application. Educating the teaching staff on how to use the mobile learning platform for assessment feedback and how to add content will help to overcome confidence issues about using a new form of technology-enhanced assessment feedback. Demonstrating to students how to use their own mobile handheld device or any device of their choice for learning will help overcome any resistance due to confidence and communication issues. Provide a demonstration for both students and lecturers, which could be achieved through regular meeting sessions where staff and students can practice using the mobile learning platform.
- Technical support – Provide more technical support to teaching staff than they usually receive. Facilitate use of the mobile learning platform from the very beginning by allowing the teaching staff to experiment with it.

**Fig. 2:**
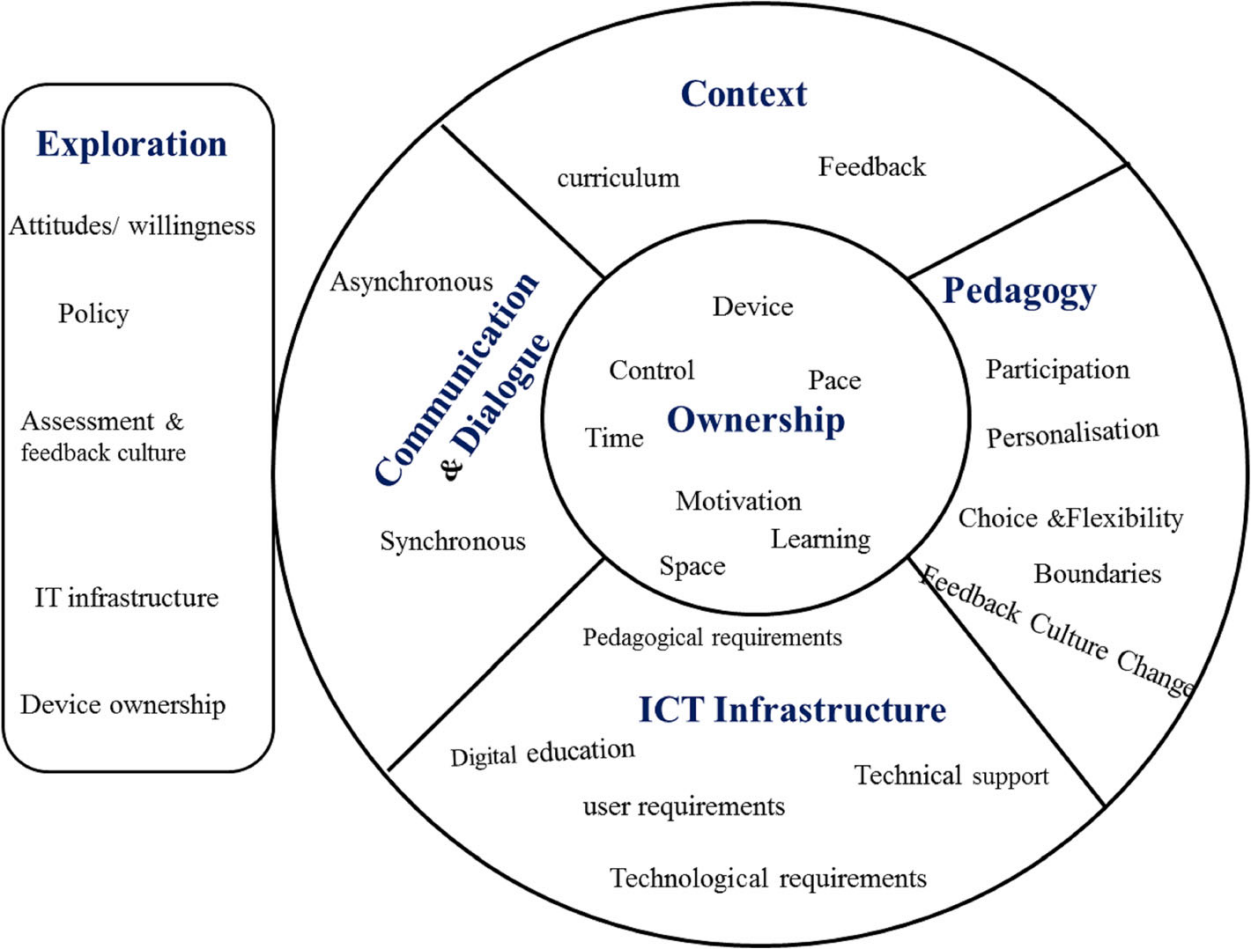
Mobile Learning Framework for Assessment Feedback based on literature review, fact-finding studies and application development and early trials

Figure 2 presents the Mobile Learning Framework for Assessment Feedback based on the literature review, fact-finding studies, and application development and early trials.

### The iterative cycles of evaluation, reflection and revision of MLFAF

**Fig. 3:**
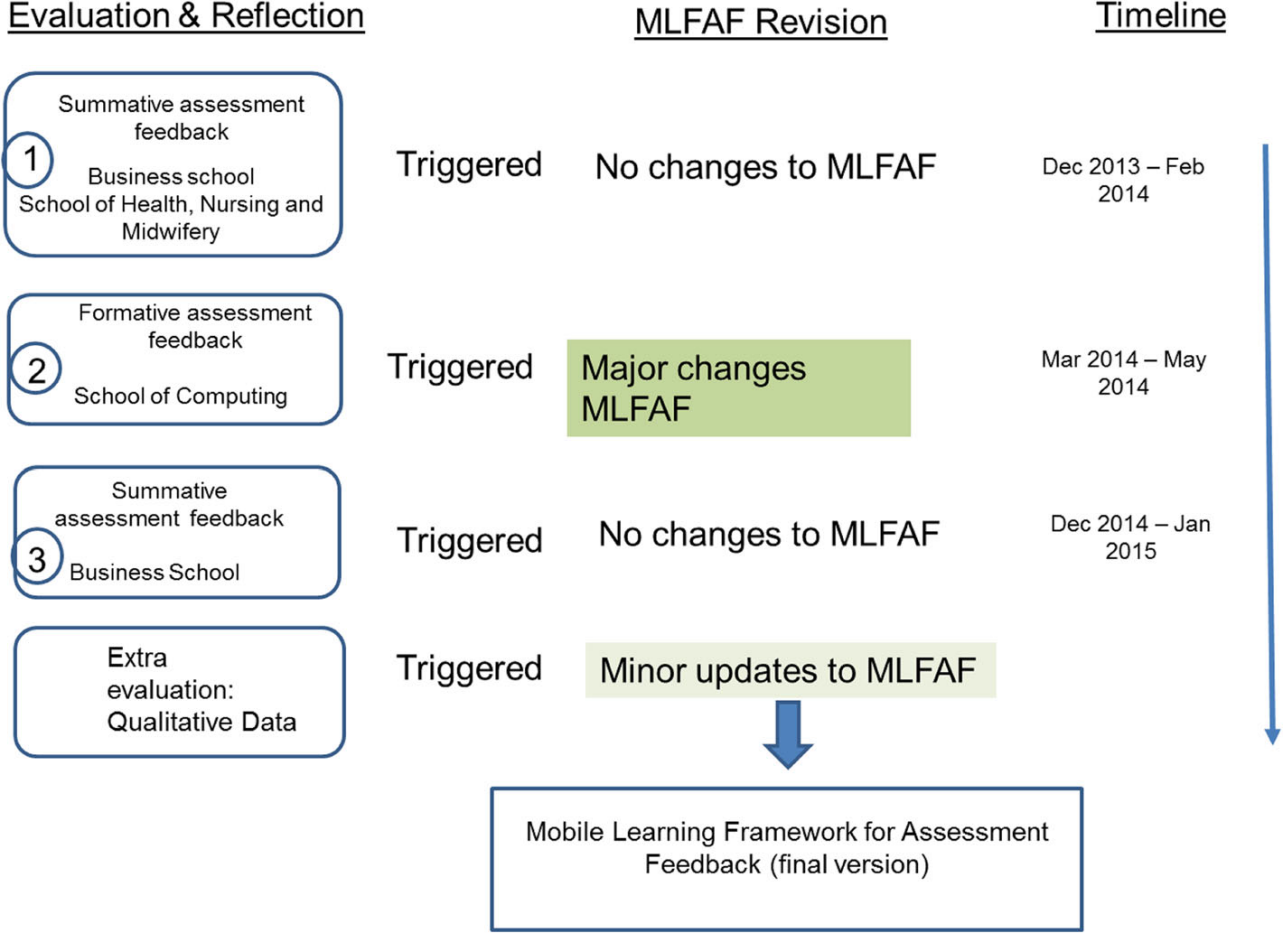
Graphical depiction of the iterative cycles of evaluation, reflection and revision of the Mobile Learning Framework for Assessment Feedback (MLFAF)

Corresponding to phase 3 of GMDR, ‘evaluation and reflection’, the purpose of this section is to evaluate and further develop the framework. The objective is to explore how the dimensions in ownership (e.g. device, learning, pace, space, time, motivation, control, engagement), context (e.g. feedback, curriculum), communication and dialogue (e.g. asynchronous, synchronous) and pedagogy (e.g. feedback personalisation, choice and flexibility) emerged in the studies and how they related to each other. The revision of the framework, where applicable, was based on the findings from the observation of the three studies and the qualitative data acquired from participants. Figure 3 presents a graphical description of the iterative cycles of evaluation, reflection and revision of MLFAF.

#### MLFAF evaluation: Study one—summative assessment feedback

The evaluation in study 1involved summative assessment feedback in the Schools of Health, Nursing and Midwifery (HNM) and Business (*n* = 218). Group assessment results were uploaded by the lecturers who would subsequently update individual student feedback and marks/grade to reflect their contribution where applicable. Using MyFeedBack to provide assessment feedback meant that students were able to access relevant feedback in authentic contexts. Students received their assessment feedback at the end of term 1, just at the beginning of their Christmas holiday (2013), implying that the feedback was accessed mostly out of the university. Students also accessed their feedback using their own devices, and many used their mobile handheld devices (smartphones and tablets) and sometimes a combination of any device. This enabled them to be in control and gave them the choice and flexibility to use a device of their choice. Log details in the database showed that access to feedback was done at any time of the day and night. Most students accessed the same assessment result on several occasions. Results showed the importance of having a tool that enables communication and feedback dialogue between students and lecturers through any device, as there was an increase in communication between the lecturer and students (Bikanga Ada 2014c). Some participants needed further help after the initial demonstration, highlighting the importance of technical support and digital education. It is evident that the potential of the aspects of the framework were achieved at this stage. It was therefore not necessary to update the framework. However, it also highlights the need for strong technical support and demonstration.

#### MLFAF evaluation: Study two—formative assessment feedback

Study 2 involved participants from the School of Computing (*n* = 79). Although students were able to access their formative feedback in authentic contexts using any device, many participants in this group did not disclose the devices they used. Compared to study 1, this study had the highest number of students accessing their feedback, but most did it just once (Bikanga Ada and Stansfield 2017). Thus, engagement with assessment feedback, though still evident, was low. This study was significantly different from study 1, in that the communication and feedback dialogue that was observed as one success factor was now non-existent in study 2. The lecturer’s participation was limited to just posting the group feedback to students. Although the feedback reached individual students on their own page where they could read it in their own privacy, it was not personalised, which could have undermined student engagement with their formative assessment feedback. It was evident that the potential of all the elements of the framework was not achieved; some dimensions of the pedagogy aspect were missing. Study 2 supported some aspects and interrelationships of the framework but failed to initiate and enhance communication and feedback dialogue.

The importance of pedagogy and pedagogical practices was evident in this study, giving a new direction to the framework. The ownership aspect, which had been at the core of the framework since the initial draft, was now replaced by the pedagogy aspect. With pedagogy at the core of the framework, its relationship with the other components of MLFAF is reciprocal and mutually influential. The ownership aspect does not necessarily lead to the communication and feedback dialogue aspect even when an ICT infrastructure that leverages the dimensions in the ownership aspect is available, as found in study 2. It is the pedagogical practices that influence communication and dialogue, facilitated by an appropriate ICT infrastructure. Ownership, however, links to pedagogy, as the pedagogical practices should ensure that the dimensions of ownership such as time, pace, space, control, device and learning are taken into consideration. In other words, considering the students as pivotal factor shapes the pedagogical practices. Pedagogy alone does not influence communication and dialogue. It needs the means by which this can be achieved (ICT infrastructure). On the other side, pedagogy affects the choice of infrastructure.

**Fig. 4:**
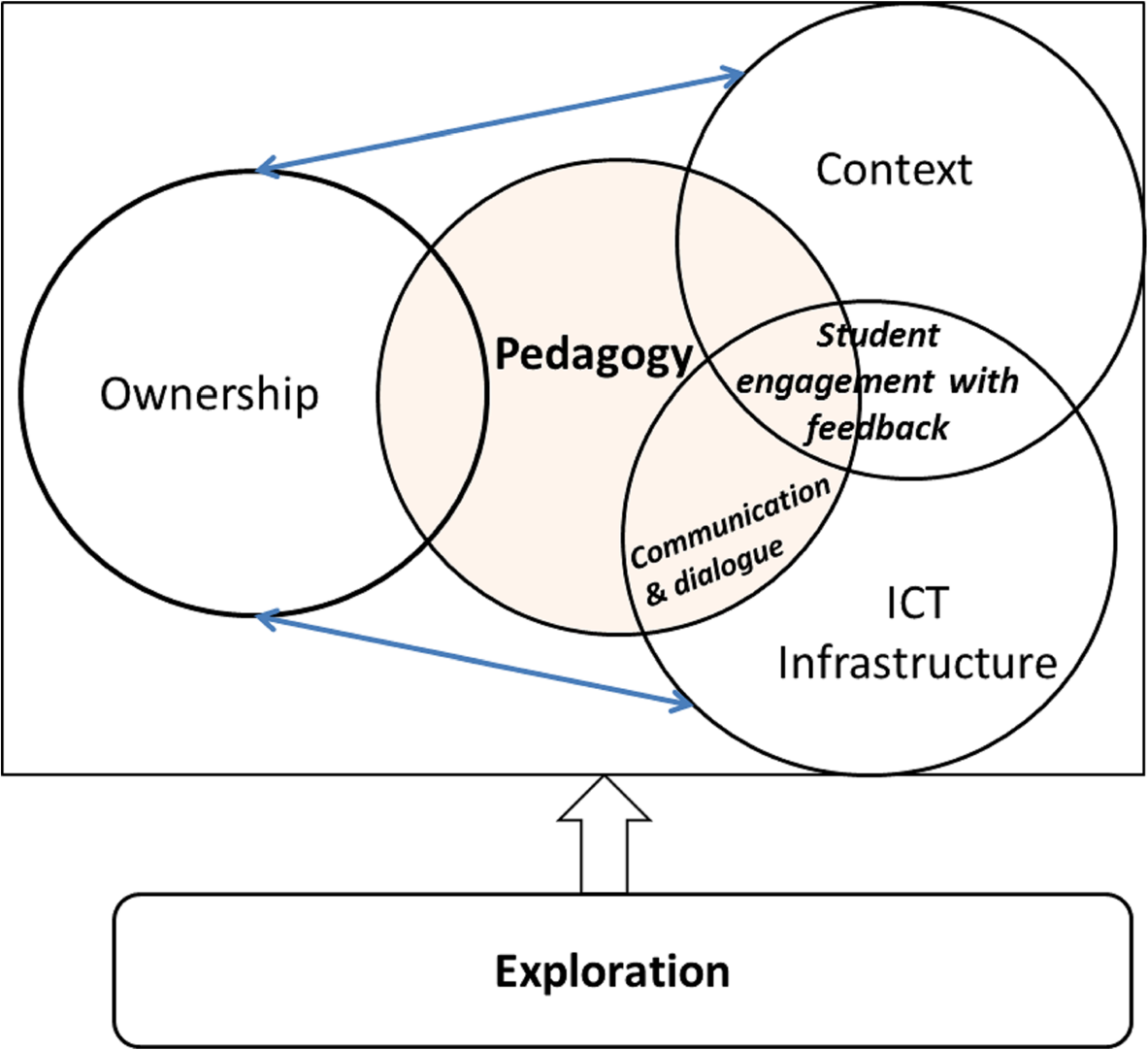
Mobile Learning Framework for Assessment Feedback based on study 2

Figure 4 shows the revised framework which now has pedagogy as the core component. It shows that student engagement with their assessment feedback happens at the intersection between pedagogy, context and ICT infrastructure (*PCI*), while communication and feedback dialogue occurs at the intersection between pedagogy and ICT infrastructure (*PI*). Meanwhile, ownership fosters access to feedback in authentic contexts. The relationship between ownership and ICT infrastructure is also reciprocal and mutually influential.

#### MLFAF evaluation: Study three—summative assessment

The newly revised framework, which puts pedagogy at the core, was further tested in study 3 (summative assessment feedback) with participants from the Business School (*n* = 148). The contexts were similar to that in study 1. The lecturer was one of those who participated in study 1. Success from the previous year had motivated her, and so, she decided to use MyFeedBack for the same group report assessment but with a different student cohort. Similar to study 1, the potential of the components of the framework was achieved, and the same conclusions were drawn. However, there was an increase in student engagement that was linked to the increase in the lecturer’s active participation on MyFeedBack, reinforcing the central place of pedagogical practices in the framework.

#### MLFAF evaluation: Qualitative data

Data from students emerged from the additional comments students left when they took the survey after using MyFeedBack application. It reinforced the place of the elements of the framework as already discussed in the studies. However, few students highlighted the difficulty in using MyFeedBack (technical support/digital education). Another issue that emerged was the timing of the feedback. Although feedback was quickly provided and was accessible anytime and anywhere using any device, some students felt that they were too busy with their exam preparation to really engage with it. Lecturer *Amina* (summative studies 1 and 2) stated that using MyFeedBack has changed the way she provides feedback and has empowered her students. Furthermore, she said that she has become more motivated as a result of her students’ engagement with assessment feedback and the dialogic feedback channels that emerged.

**Fig. 5:**
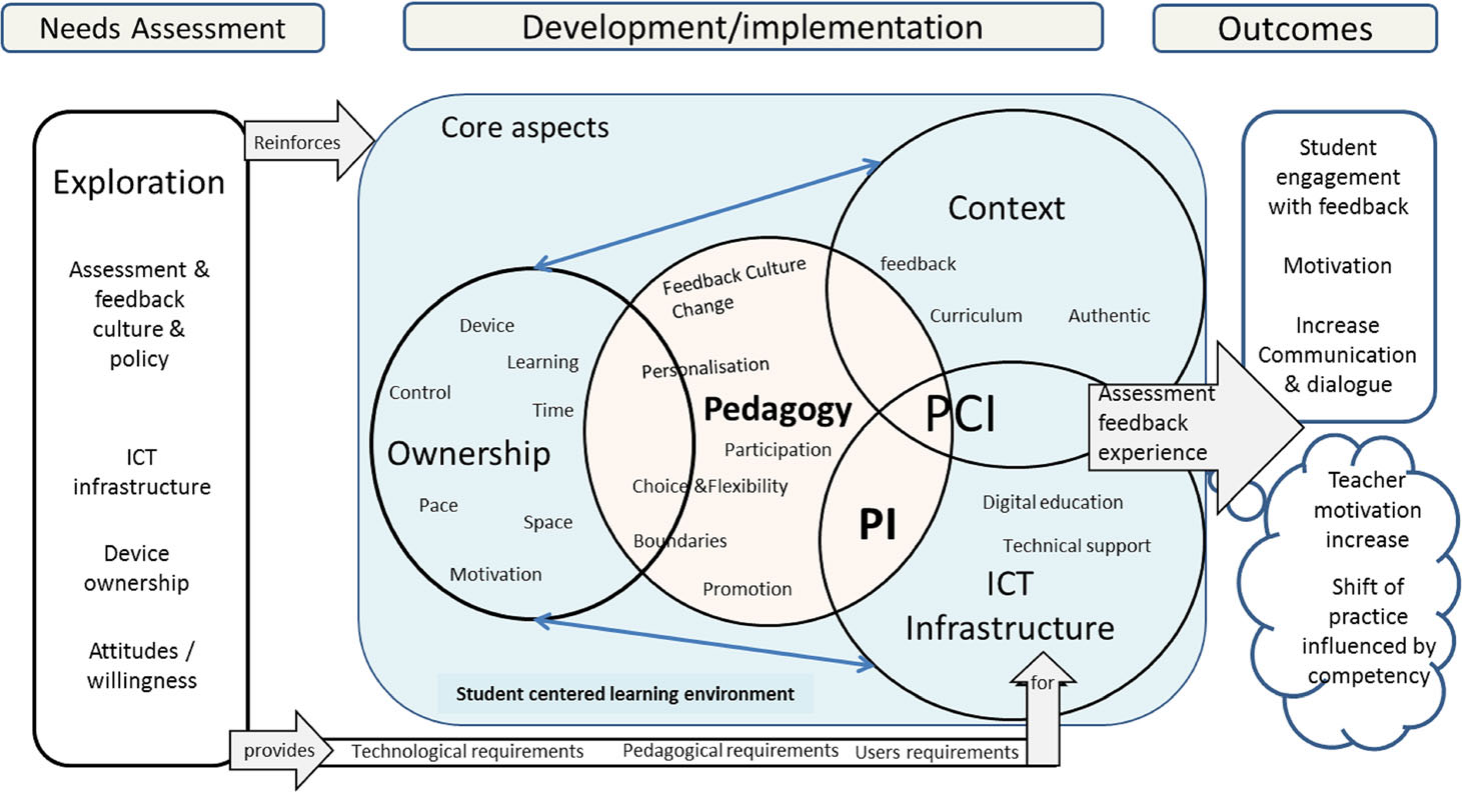
Mobile Learning Framework for Assessment Feedback

The framework was then revised to highlight the fact that student engagement with the feedback influences the shift in the lecturer’s pedagogical practices and increases the lecturer’s motivation. Moreover, the change in pedagogical practices is influenced by the lecturer’s own competency. Figure 5 presents the final version of MLFAF, with all the components organised in three main sections: Needs assessment, Development/implementation and Outcomes. The proposed framework is an answer to a part of the research question that asks what framework educators should follow when creating mobile learning initiatives.

### How to use MLFAF for supporting assessment feedback?

MLFAF is mostly useful in situations where students’ lack of engagement with their assessment feedback is evident and/or students are not commenting on the received feedback. Both issues often leave educators frustrated and disillusioned. MLFAF should be used as a stepping stone to ask all the correct questions, gather information, critically examine what is needed and what is happening, and reflect on what might need to be changed. MLFAF can also be used in other mobile learning activities. It is an ideal foundation to develop wide-scale mobile learning initiatives that underpin longitudinal practices as opposed to short-term formal/informal practices. It means adopting these practices for long term and making them the norm by including them in the curriculum instead of trying them once or twice and then abandoning them. MLFAF has three main sections: Needs assessment, Development/Implementation and Outcomes. Table 1 presents an example of a decision-making exercise that could guide the practitioners.

**Table 1:** Example of decision-making exercise using MLFAF

Section	Needs assessment	Ask questions	How to achieve that?
Aspect			
Exploration	A preliminary investigation is required for any mobile learning initiative.	Is there an ICT infrastructure to support the mobile learning activity? What does the policy say? What is the current culture? What type of devices do students own? What do students (and educators) think of the mobile learning activities? Will they be willing to participate? How do I foster the Use My Own Device attitude?	Meeting with institution’s ICT personnel to identify what enabling technology is available. If none, develop one. Check policy document and assessment and feedback practices. Consider surveys and interviews
Section	Development and implementation	Ask questions	How to achieve that?
Aspect			
Ownership	Foster student ownership of the learning and of the device.	Are my learning activities accessible using any device? Can I enable online and/or offline access?	Produce device-agnostic learning material. Depending on available technology
Pedagogy	Any activity that would improve student learning.	How do I change the feedback culture? How do I promote the activity? What boundaries shall I set? How will I manage my time? What personal challenges will I face?	Online information and training/seminars Active participation form lecturer is important. Tell students when you will be available on the mobile learning platform.
ICT infrastructure	Appropriate infrastructure to provide and support mobile learning.	How will technical support be made available? What digital education measures should be put in place?	Produce online training and ‘How to’ pages. Have regular seminars/training.
Context	Of the activities	Is the mobile learning activity relevant? Does it supplement or replace any current activity? How can I incorporate the activity into the curriculum as part of my regular teaching process?	Ensure it is linked to real student work. Learners are busy and may find mobile learning less attractive if it is not linked to their curriculum.

## Design guidelines for mobile learning assessment feedback

This section answers part of the research question that asks about the design principles educators should follow in creating mobile learning initiatives.

The key elements and characteristics of the MLFAF framework are exploration, pedagogy, context, ICT infrastructure and ownership. The MLFAF framework has enabled the compilation of design considerations and implementation guidelines, which are as follows:

### Exploration

Practices of assessment feedback are different between individuals and between institutions. Exploration helps with the identification of the issues, assumptions and beliefs of stakeholders that affect the area of mobile learning undertaken. It is concerned with an initial fact-finding investigation that allows the understanding and specification of the context, the user requirements and the objectives in order to influence the design of the artefact. It enables looking into the current practice of assessment feedback, looking into the policy and the institution’s culture, and investigating students’ attitudes and the devices they own or use. It is also important to investigate the current state of technology available.

This aspect will help the practitioners set firm foundations for the mobile learning initiative, including the design and development requirements of a mobile application, if applicable. The guidelines related to the preliminary investigation include the following:

- Define firm foundations for the assessment feedback strategies.

Investigations should cover issues related to assessment and feedback culture, and policy and ICT infrastructure because these elements either support or affect the provision of assessment feedback.

- Pay attention to lecturers and students’ issues, their devices and attitudes.
- Define foundations for ICT infrastructure that leverages the use of student-owned devices.

### Pedagogical considerations and context guidelines

Pedagogy is about the practices that influence the learning experience and, particularly, the assessment feedback experience. Context is about access to feedback anywhere, in authentic contexts, ensuring that the content is relevant, and thus, the need to embed mobile learning activities within the curriculum becomes crucial.

This section answers the part of the research question that asks: ‘What pedagogical strategies can best be deployed to enhance student engagement with their assessment feedback and foster dialogic feedback communication channels between educators and students?’

In order to foster student engagement with feedback and enhance communication,

- Pay attention to the interaction and participation. As observed in this study, the interaction with the mobile web application results in a positive outcome when both lecturers and students participate.
- Enable choice and flexibility. Enable participants to make their own choices (anytime, anywhere and on any device).
- Provide personalised assessment feedback, which students prefer. Ensure the teaching and learning is tailored to individual needs. Provide the facilities that enable personalisation of student feedback.
- Provide pedagogical support and training so that educational technologies can be integrated into the lecturers’ practices and embedded within the institution’s learning management systems.
- Integrate the mobile learning assessment feedback practices into the curriculum and ensure the relevance of the mobile learning activities. An attempt to provide mobile learning activities that are not directly related to the curriculum will fail.
- Foster feedback culture changes by educating students on feedback prior to implementing the mobile learning strategy, which will help to enforce the idea behind the mobile learning strategy and the importance of students’ participation in a feedback dialogue.
- Educators should reinforce the importance of feedback for student learning enhancement and subsequently to the institution as a whole.
- If you want to enable student engagement with their feedback, you are best advised to enable the assessment feedback in authentic contexts. These authentic contexts can be defined by the anywhere, anytime and using any device affordances that are brought by ownership. Feedback should be accessible in a formal and informal environment.
- The assessment feedback provided must be in context.
- Enable fostering of cultural changes by holding regular meeting sessions where staff and students can practice using the mobile learning platform. This will aid the teaching staff to overcome their fear of using the mobile learning platform or a new technology and encourage students to use their own devices.
- Measure the real, as opposed to the perceived, impacts of new technologies by considering digital footprinting, as seen in this study.
- Evaluate students’ and educators’ opinions of the feedback delivery method. This ensures that the relevancy of the learning activities has been established.
- Evaluate students: This will inform whether the educational goals are achieved. For example, engagement with feedback and communication.
- Provide opportunities to share new practices with guidelines that provide direction and reassurance to staff about the assessment feedback practices. Promoting the benefits of using mobile learning for assessment feedback among the teaching staff is crucial for obtaining educators’ acceptance and adoption of mobile learning.
- It is important to promote the benefits at the institution’s conferences, as well as formal and informal meetings. In this study, the researcher attended several Teaching and Learning conferences organised by the university where the benefits of using MyFeedBack for assessment feedback were presented via posters. During the conference in June 2014, both the researcher and the main lecturer did a joint presentation entitled ‘Using MyFeedBack mobile Web 2.0 system to understand the conundrum of unknown students’ behaviour upon receipt of their assignment results’. The lecturer shared her experiences of reigniting assessment feedback engagement and dialogic feedback channels using MyFeedBack. This resulted in further one-to-one meetings with other lecturers interested in providing assessment feedback using an enabling mobile learning platform.

### Ownership

Ownership is about students reclaiming control over the devices that suit their taste or convenience, along with the time they want to access their content, the place they want to access that content and the pace at which they interact with it. It is about student motivation and being in control of their choices in a student-centred environment. With regard to this aspect, the following should be taken into consideration:

- Enable student control of learning in a student-centred environment by creating device-agnostic activities.

### ICT infrastructure

An appropriate ICT infrastructure is necessary as it will allow learners to be in control and have the choice and flexibility to access the learning content anywhere, anytime and using any device, which, subsequently, can increase communication and dialogue. This is also about the provision of digital education and technical support to lecturers and students. The design and implementation guidelines for the ICT infrastructure are as follows:

With regard to the design of the mobile web application for assessment feedback, the following should be taken into consideration:

- Ground the design of mobile learning platform for assessment feedback on insights gained from the results of the initial explorative studies.

With regard to the actual development of the mobile web application for assessment feedback, the following should be taken into consideration:

- If you want to design a mobile learning environment or appropriate the available ICT infrastructure for mobile learning assessment feedback, then you are best advised to design an enabling platform that will allow wide participation by letting students use any device of their choice, including their smartphones.
- When introducing similar technology into educational settings, information about the technical requirements that would enhance its use must be explicitly provided to lecturers and students.
- Any changes in the functionality should be regularly reported, the usability of the application should be evaluated on several occasions in order to improve it and the use of a mobile web application to cater to the diversity in student device and their choice should be considered.

With regard to the implementation of the mobile web application for assessment feedback, the following should be taken into consideration:

- Maintain a constant interaction with the participants. This will increase the possibility of lecturers using the application and getting familiarised with its features.
- Educating the teaching staff on how to use the mobile learning platform for assessment feedback and communication will help to overcome confidence issues in the teaching staff about using a new form of technology-enhanced assessment feedback.
- Educate students on how to use their own mobile handheld device or any device of their choice for assessment feedback in order to overcome any resistance due to confidence and communication issues.

## Conclusions and future work

This study contributes to the body of knowledge on assessment feedback in mobile learning through the development of a Mobile Learning Framework for Assessment Feedback (MLFAF). The framework highlights the importance of pedagogy and pedagogical practices as the core aspect of an environment that fosters the use of student-owned devices for assessment feedback. MLFAF is designed to provide educators, policy-makers and researchers with a representation of how student devices could be harnessed in the context of assessment feedback in order to increase their engagement and foster communication and feedback dialogue with their lecturers. It also provides a broader view that takes into account the key aspects to be considered, which are likely to succeed or may not succeed in any mobile learning implementation.

Practitioners are encouraged to consider using MLFAF to plan, develop and evaluate longitudinal mobile learning initiatives in different environments and in different disciplines. Further research might help to identify and explore the factors influencing engagement with the summative and formative assessment feedback, and how, or if, these affect the dynamics of the components of the framework.
